# Cognition, function, and mood post-COVID-19: Comparative analysis using the health and retirement study

**DOI:** 10.1371/journal.pone.0315425

**Published:** 2024-12-18

**Authors:** Han Su, Pei-Lin Yang, Tammy L. Eaton, Thomas S. Valley, Kenneth M. Langa, E. Wesley Ely, Hilaire J. Thompson

**Affiliations:** 1 School of Nursing, Vanderbilt University, Nashville, Tennessee, United States of America; 2 School of Nursing, National Defense Medical Center, Taipei, Taiwan; 3 Veterans Affairs Health Services Research & Development, Center for Clinical Management Research, Veterans Affairs Ann Arbor Healthcare System, Ann Arbor, Michigan, United States of America; 4 Division of Pulmonary and Critical Care Medicine, Department of Internal Medicine, U-M, Ann Arbor, Michigan, United States of America; 5 Institute for Healthcare Policy and Innovation, U-M, Ann Arbor, Michigan, United States of America; 6 Department of Internal Medicine and Cognitive Health Services Research Program, U-M Medical School, Ann Arbor, Michigan, United States of America; 7 Institute for Social Research, U-M, Ann Arbor, Michigan, United States of America; 8 Critical Illness, Brain Dysfunction and Survivorship Center, Vanderbilt University Medical, Nashville, Tennessee, United States of America; 9 Division of Allergy, Pulmonary and Critical Care Medicine, Vanderbilt University Medical Center, Nashville, Tennessee, United States of America; 10 Veterans Affairs Tennessee Valley Geriatric Research Education Clinical Center, Nashville, Tennessee, United States of America; 11 School of Nursing, University of Washington, Seattle, Washington, United States of America; 12 School of Medicine, University of Washington, Seattle, Washington, United States of America; Indian Institute of Technology Jodhpur, INDIA

## Abstract

Millions of Americans endure post-COVID conditions (PCC), yet research often lacks pre-illness measurements, relying primarily on follow-up assessments for analysis. The study aims to examine the prevalence of PCC, including cognitive impairment, functional limitation, and depressive symptoms, along with relevant risk factors, while controlling for individuals’ pre-illness status measured in 2018. A cross-sectional retrospective study utilized the 2018 and 2020 Health and Retirement Study surveys. Sample included individuals with COVID-19 (n = 409; average age 64) and individuals without COVID-19 (n = 8689; average age 59). COVID-19 positive: Individuals with positive tests, physician diagnoses, emergency room visits, or hospitalizations for COVID-19 between 2019–2020. Cognition was assessed using immediate and delayed word-recall tests, serial seven subtractions, and backward counting. Functional status was measured using limitations in activities of daily living (ADLs) and instrumental ADLs. Depressive symptoms were measured using the modified Center for Epidemiology Studies Depression scale. Participants’ perception of experiencing PCC was collected. Logistic regression and propensity score matching were employed for these analyses. Among 409 COVID-19-positive respondents (14% hospitalized), 24% exhibited new impairments after COVID-19 infection. Noteworthy increases in functional limitation (OR [95% CI]: 2.18 [0.95, 0.97], p < 0.001) and a marginal rise in cognitive impairment (1.79 [0.99, 3.32], p = 0.053) following COVID-19 infection were observed in comparison to their pre-COVID-19 baseline. Compared to 8689 non-COVID-19 cases, the 409 COVID-19 positives showed increased functional decline (1.78 [1.26, 2.51], p = 0.001) and depressive symptoms (1.41 [1.04, 1.91], p = 0.03). Factors associated with PCC included pre-existing impairments, lower education, female gender, prior hospitalization, higher comorbidity, lower wealth, and mild COVID-19. A notable number of respondents, especially older individuals with fewer pre-existing health conditions, experienced PCC without awareness. Compared to an individual’s pre-illness baseline and uninfected individuals, being positive for COVID-19 raised the risk of functional limitation, depressive symptoms, and cognitive impairment. Additionally, addressing PCC through both subjective and objective approaches is essential to alleviate individual and societal burdens.

## Introduction

The COVID-19 pandemic has affected more than 770 million people worldwide since the end of 2019 [1]. Nearly 45% of COVID-19 survivors experience lingering symptoms and disabilities following infection, known as post-COVID conditions (PCC) [2–4]. The Centers for Disease Control and Prevention (CDC) defines post-COVID conditions as new, returning, or ongoing health problems occurring ≥4 weeks after being infected with SARS-CoV-2. The prevalence of PCC and the specific occurrence of various PCCs have been well-documented across multiple studies. For instance, the prevalence of depressive symptoms 12 weeks or more after infection ranges from 11% to 28%, with clinically significant depression or severe depressive symptoms reported in 3% to 12% of individuals [5]. Fatigue is observed in approximately 32% of individuals beyond 12 weeks post-COVID-19 diagnosis, while cognitive impairment affects an estimated 22% [6]. These ongoing health problems can significantly hinder the resumption of daily activities, reduce quality of life, increase healthcare expenditures, and diminish productivity [3, 7, 8].

Despite the considerable impact posed by PCC, the prevalence of this condition remains undetermined. Existing studies have mainly described the prevalence of PCC at follow-up but are unable to compare symptoms and disabilities to pre-infection baseline (cognition, functional limitation, and depressive symptoms) [9–12]. This presents a significant concern, as research indicates that individuals with pre-existing conditions such as anxiety, depression, and disability are disproportionately affected by both COVID-19 and PCC [13–17]. Furthermore, studies investigating PCC frequently lack a proper comparison group [18–20]. As a result, the relationship between COVID-19 infection and observed outcomes remains unclear. It is imperative to gain a comprehensive understanding of the prevalence of PCC and identify the risk factors associated with this condition. This knowledge will play a crucial role in effectively identifying and managing PCC.

This study aims to address the existing knowledge gap by conducting pre- and post-COVID-19 comparisons, as well as a comparative analysis between COVID-positive and COVID-negative individuals, with a focus on cognitive impairment, functional limitations, and depressive symptoms, utilizing data from the Health and Retirement Study (HRS). Furthermore, the study seeks to identify factors associated with the exacerbation of these conditions among individuals affected by COVID-19.

## Methods

### Data source

The HRS, sponsored by the National Institute on Aging (grant number NIA U01AG009740) and conducted by the University of Michigan, is a biennial longitudinal study of over 20,000 Americans aged 51 and older, along with their spouses, without age restrictions. Since 1992, the study has collected data regarding income, health insurance, physical health, and cognitive function in respondents.HRS divided the sample into face-to-face and telephone interviews. However, due to COVID-19, all interviews were conducted by telephone, and a COVID-19 module was introduced in 2020, administered to half of the households originally designated for face-to-face interviews. For this analysis, we studied all respondents who completed the HRS 2020 COVID-19 module and interviews conducted in 2018 and 2020 in which cognition, function, and depressive symptoms were evaluated. Ethics Statement: Since the data were deidentified and publicly accessible, IRB approval was not necessary for this analysis.

### Social demographic and pre-COVID-19/baseline health conditions

Specific demographics and clinical data (age, sex, race, education, marital status, health insurance, total wealth, number of health conditions, and hospitalization between 2018 and 2020) were extracted from the RAND HRS longitudinal file 2020. The number of health conditions is the sum of self-reported doctor-diagnosed: high blood pressure; diabetes; cancer of any kind except skin cancer; heart problems; emotional or psychiatric problems; arthritis; dementia or Alzheimer’s disease. Total wealth is calculated as the sum of all wealth components (i.e., the net value of real estate, Individual Retirement Accounts, checking, and savings), less all debt (i.e., home loans).

### COVID-19 history

COVID-19-related variables were extracted from the COVID-19 module of the 2020 HRS survey. COVID-19 history is dichotomized as "positive" or "negative." We defined COVID-19 positive if the respondent answered "Yes" to one of the below questions: "Have you had COVID," "Tested positive for COVID," "Doctor confirmed COVID," "Visited emergency room due to COVID" or "Admitted to a hospital due COVID." Other COVID-19-related variables were also collected, such as positive COVID-19 test year and month, nights in the hospital for COVID-19, and whether participants perceived themselves as having PCC (“Have you experienced any lingering physical or mental health effects from the virus?”). For individuals with COVID-19 positive, we calculated the time since COVID-19 infection using the time of the HRS 2020 survey completed less the time of the positive COVID-19 test.

### Pre- and post-COVID-19 symptoms and disabilities measurements

We defined measurements collected in 2018 as pre-COVID-19/baseline and those collected in the 2020 survey as post-COVID-19/follow-up. Cognition was extracted from 2018 and 2020 HRS Core data and assessed using a 27-score validated battery that included immediate and delayed word-recall tests, serial seven subtractions, and backward counting. Cognitive impairment was defined as a total score of less than 12 [21, 22]. Functional status and depressive symptoms were extracted from the RAND HRS longitudinal file 2020. Functional status was measured using six activities of daily living (ADLs: walking, dressing, bathing, eating, getting into and out of bed, and toileting) and five instrumental ADLs (IADLs: preparing a hot meal, shopping for groceries, making telephone calls, taking medicines, managing money). A sum of ADLs and IADLs, with a score ≥1, was defined as having functional limitation [23]. Depressive symptoms were measured using the modified 8-item Center for Epidemiology Studies Depression (CESD) scale [24], with scores ≥4, defined as having high depressive symptoms [25]. We further defined self-awareness of PCC as participants who develop new cognitive impairments, physical limitations, or high depressive symptoms after a COVID-19 infection, as previously defined, and perceive themselves as having PCC.

### Analyses

We summarized data using median and interquartile range (IQR) for continuous variables and proportions for categorical variables. Bivariable logistic regressions were conducted to examine the association between demographic, pre-COVID-19/baseline number of health conditions, pre-COVID-19/baseline cognitive impairment, functional limitation, and depressive symptoms, as well as COVID-19 infection (positive, negative). All analyses were performed using Stata statistical software (StataCorp, College Station, TX, USA) and R (R Development Core, Vienna, Austria). A two-sided P < .05 denoted statistical significance.

For the primary objective, we used the McNemar test among individuals with a positive COVID-19 diagnosis to determine if there were differences in cognitive impairment, functional limitation, and high depressive symptoms before and after COVID-19. Then, we conducted multivariable logistic regression models in individuals with a positive COVID-19 diagnosis to identify factors associated with worsened cognition, functional ability, and high depressive symptoms after COVID-19 infection.

For the third objective, multivariable logistic regression models were used to estimate the association between COVID-19 diagnosis (positive versus negative) and post-COVID-19/follow-up cognitive impairment, functional limitation, and high depressive symptoms, separately, controlling for pre-COVID-19/baseline conditions. Initial multivariable logistic regression models adjusted demographics, hospitalization between 2018 and 2020, pre-COVID-19/baseline health conditions, and pre-COVID-19/baseline cognitive impairment, functional limitation, or depressive symptoms.

To evaluate the robustness of the results, we conducted an analysis using propensity-score matching to reduce confounding effects in the study [26, 27]. Propensity scores were calculated using multivariable logistic regression for cognitive impairment, functional limitation, and high depressive symptoms model separately. For each model, the propensity score included variables that may impact both COVID-19 history and outcomes (post-COVID-19/follow-up cognition, functional limitation, or depressive symptoms) and were unbalanced between exposure groups. Variables included were demographic, hospitalization between 2018 and 2020, number of health conditions at pre-COVID-19/baseline, and pre-COVID-19/baseline cognition, functional limitation, and depressive symptoms. Association between COVID-19 history (positive versus negative) and post-COVID-19/follow-up cognitive impairment, functional limitation, and high depressive symptoms were then estimated by multivariable logistic regression models using propensity score matching. Matching was performed based on propensity scores using a 1:1 nearest-neighbor algorithm without replacement with a caliper width of 0.05. The best-matched cohort was identified based on the most balanced distribution of propensity scores and the best balance in individual covariates between groups (COVID-19 positive versus negative).

## Results

**Fig 1:**
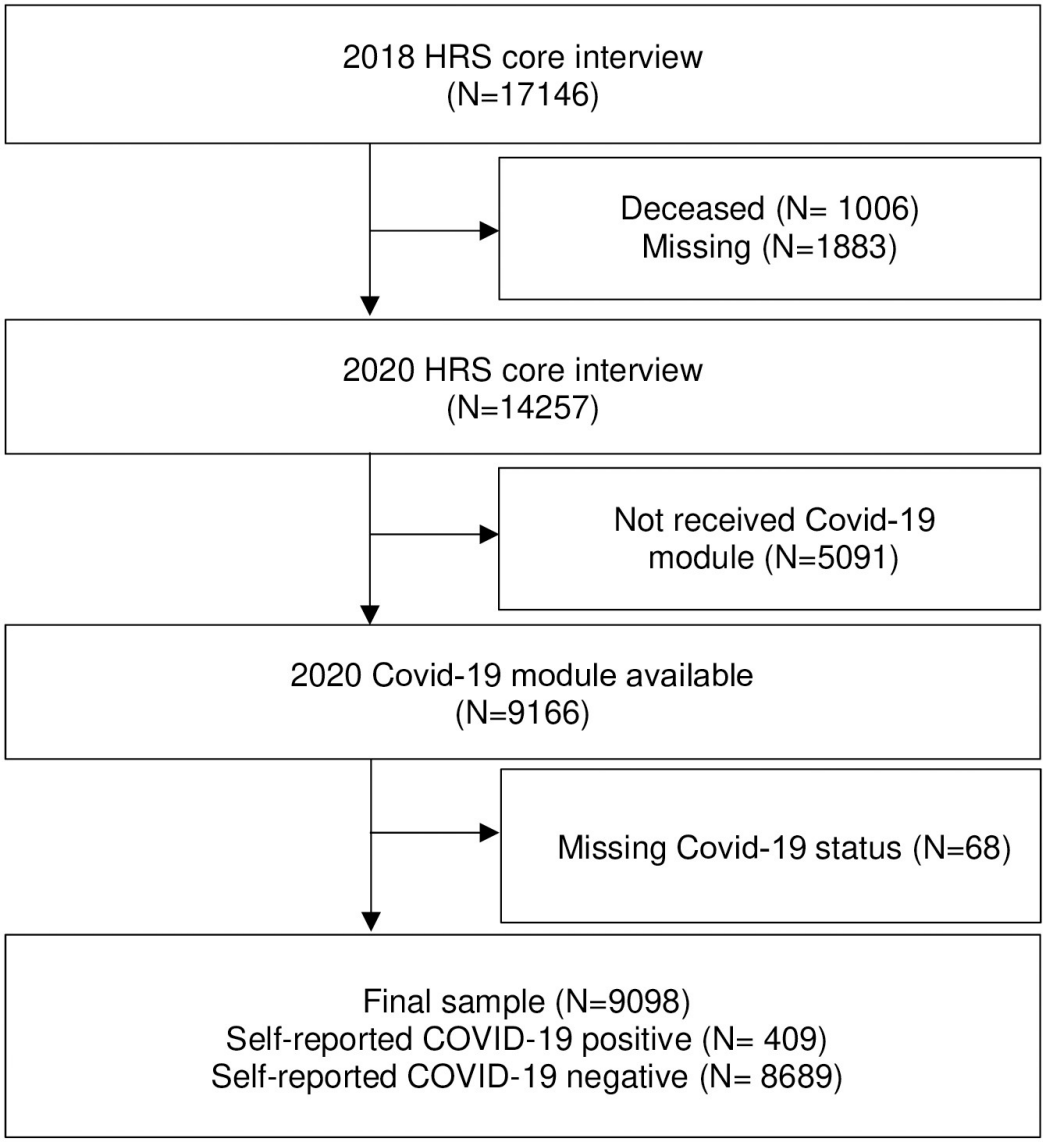
Patients flow chart. Abbreviation: Health retirement stud = HRS.

The analyses encompassed 409 COVID-19-positive cases and 8689 COVID-19-negative cases (Fig 1). Demographic and baseline cognition, function, and depressive symptoms are summarized in Table 1, both in unmatched and propensity-score–matched analytic samples. In the unmatched sample, as compared to respondents who reported being negative for COVID-19, those who reported being COVID-19 positive tended to be younger (odds ratio [OR] [95% confidence interval (CI)]: 0.96 [0.95, 0.97], p<0.001), non-White (0.71 [0.5,9 0.85], p<0.001), and experiencing functional limitation at baseline (1.38 [1.05, 1.78], p = 0.02, eTable1). As compared to respondents without any health insurance, those with federal insurance only (0.44 [0.32, 0.62], p<0.001) or both federal and private insurance (0.45[0.31, 0.67], p<0.001) were less likely to report being COVID-19 positive (eTable1). Those with a total wealth over $125,000 were less likely to report being positive for COVID-19 than those with a total wealth under $25,000 (0.73 [0.59, 0.92], p = 0.005, eTable1).

**Table 1:** Demographic, baseline cognition, function, and depressive symptoms by COVID history, before and after propensity-score matching.

Variables	Unmatched Patients*			Propensity-Score–Matched Patients								
				Cognitive Impairment Model			Functional Limitation Model			High Depressive Symptoms Model		
	COVID-19 Negative (n = 8689)	COVID-19 Positive (n = 409)	p^£^	COVID-19 Negative (n = 321)	COVID-19 Positive (n = 321)	p^£^	COVID-19 Negative (n = 385)	COVID-19 Positive (n = 385)	p^£^	COVID-19 Negative (n = 357)	COVID-19 Positive (n = 357)	p^£^
Age, Median (IQR), y	64 (17)	59 (12)	<0.001	58 (11)	58 (10)	0.90	59 (11)	59 (12)	0.80	59 (9)	58 (11)	0.80
Female, No. (%)	5,251 (60)	239 (58)	0.40	181 (56)	192 (60)	0.40	218 (57)	223 (58)	0.70	186 (52)	211 (59)	0.06
White Race, No. (%)	5,570 (64)	226 (56)	<0.001	183 (57)	172 (54)	0.40	209 (54)	216 (56)	0.60	199 (56)	197 (55)	0.90
Education, Median (IQR) y	13 (4)	13 (3)	0.11	13 (4)	13 (3)	0.60	13 (4)	13 (3)	0.20	14 (4)	13 (4)	0.13
Married or partnered, No. (%)	5312 (64)	272 (67)	0.03	227 (71)	213 (66)	0.20	260 (68)	259 (67)	0.90	249 (70)	241 (68)	0.50
Total wealth ($), No. (%)												
<25 000	2,451 (28)	121 (30)	0.01	98 (31)	100 (31	0.60	102 (26)	114 (30)	0.60	106 (30)	105 (29)	0.90
25000–124999	1,704 (20)	101 (25)		74 (23)	84 (26)		95 (25)	95 (25)		87 (24)	91 (25)	
≧125000	4,534 (52)	187 (46)		149 (46)	137 (430)		188 (49)	176 (46)		164 (46)	161 (45)	
Insurance, No. (%)												
No insurance	680 (8.0)	52 (13)	<0.001	44 (14)	43 (13)	0.70	42 (11)	51 (13)	0.10	40 (11)	47 (13)	0.40
Federal	3,653 (43)	123 (31)		89 (28)	96 (30)		101 (26)	119 (31)		107 (30)	104 (29)	
Private	2,607 (31)	165 (42)		154 (48)	142 944)		166 (43)	161 (42)		173 (48)	157 (44)	
Federal + Private	1,582 (19)	55 (14)		34 (11)	40 (12)		76 (20)	54 (14)		37 (10)	49 (14)	
Number of Health Conditions, Median (IQR.), ^‡^	2 (2)	2 (2)	0.02	2.1 (1.6)	2.1 (1.6)	0.88	2.1 (1.6)	2.2 (1.7)	0.33	2 (1.5)	2.1 (1.6)	0.40
Hospitalizations between 2018–2020, No. (%)	1,908 (22)	127 (32)	<0.001	90 (28)	94 (29)	0.70	115 (30)	122 (32)	0.60	97 (27)	107 (30)	0.40
Baseline Cognitive Impairment ^¶^, No. (%)	1,374 (19)	61 (17)	0.40	50 (16)	50 (16)	>0.90						
Baseline Functional Limitation ^§^, No. (%)	1,165 (13)	72 (18)	0.02				61 (16)	67 (17)	0.60			
Baseline High Depressive symptoms ^‖^, No. (%)	1,253 (15)	71 (18)	0.07							61 (17)	61 (17)	0.90

* Missing data (n, %): white race (41, 0.4%), years of education (47, 0.5%), marital status (17, 0.2%), insurance (181, 2%), hospitalization (78, 0.9%), cognitive impairment (1,558, 17%), high depressive symptoms (268, 0.3%)

^£^ Calculated by Student t-test for continuous variables and X^2^ or Fisher exact tests, as appropriate, for categorical variables.

^‡‡^A sum of whether the respondent has or had high blood pressure or hypertension; diabetes or high blood sugar; cancer or a malignant tumor of any kind except skin cancer; chronic lung disease except asthma such as chronic bronchitis or emphysema; heart attack, coronary heart disease, angina, congestive heart failure, or other heart problems; stroke or transient ischemic attack; emotional, nervous, or psychiatric problems; arthritis or rheumatism; Dementia or Alzheimer’s disease—range from 0 to 9.

^¶^A 27-point scale was administered that included tests of memory, serial 7 subtractions, and backward counting. Cognitive impairment is defined as a total score <12.

^§^A sum of ADLs and IADLs, with a score ≥1, is defined as physical impairment.

^‖^An 8 -points scale measured by CES-D-8, with scores ≥4, is defined as having high depressive symptoms.

### Pre-and post-COVID-19 cognition, function, and depressive symptoms

**Fig 2:**
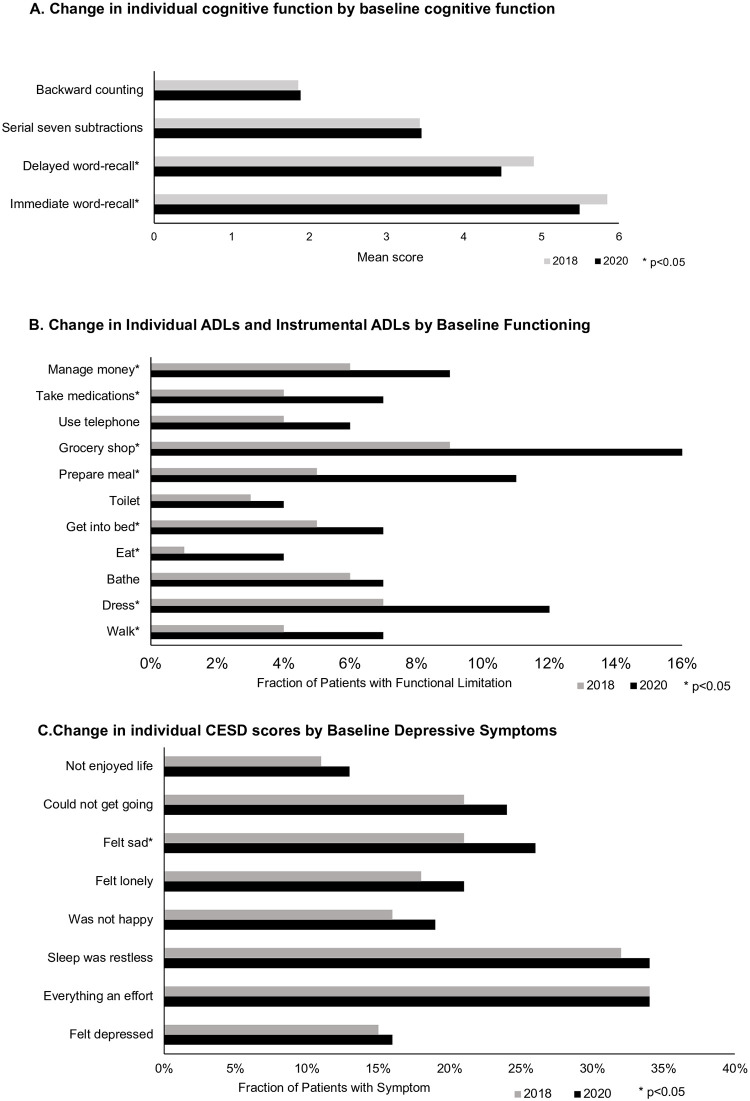
Change in individual cognition, functional limitation, and depressive symptoms by baseline functioning in COVID-19 cases.

Among the 409 respondents with COVID-19 positive, the HRS 2020 survey was completed, on average, two months after their infection. Out of these individuals, 57 were admitted to the hospital, with a median (IQR) length of stay of 4 (8) days. Mechanical ventilation was required for 9% (n = 5) of the hospitalized patients. After COVID-19 infection, a total of 24% of individuals exhibited a minimum of one new impairment, with specific proportions corresponding to distinct impairments: 12% for cognitive impairment, 9% for functional limitation, and 12% for high depressive symptoms. McNemar test revealed a marginal association with an increase in cognitive impairment (1.79 [0.99, 3.32], p = 0.053), as well as a significant increase in functional limitation (OR [95% CI]: 2.18 [0.95, 0.97], p<0.001) following COVID-19 infection when compared to their own pre-COVID-19 baseline. Memory performance was impacted, as evidenced by significant decreases in immediate word recall (from 5.85 to 5.49, p = 0.001) and delayed word recall (from 4.90 to 4.48, p<0.001, Fig 2). The functional impairment affected various ADLs and IADLs (Fig 2). However, there was no significant difference in the prevalence of high depressive symptoms before and after COVID-19 infection (1.52 [0.93, 2.51], p = 0.10).

Out of the 313 respondents who tested positive for COVID-19 and underwent cognitive battery, ADL/IADL, and CESD assessments, as well as responded to queries regarding their perception of having PCC, 38% (n = 119) reported experiencing PCC after the infection. Among the 313 respondents, 89 developed new cognitive impairment, functional limitations, and/or high depressive symptoms post-infection. Interestingly, approximately half of them (n = 49) were unaware of these emerging impairments. Multivariable logistic regression analysis demonstrated that older age (OR [95% CI]: 1.07 [1.02, 1.13], p = 0.006) and fewer pre-existing health conditions prior to COVID-19 (0.72 [0.54, 0.95], p = 0.02) were associated with the lack of awareness regarding PCC.

### Factors associated with PCC

Respondents with cognitive impairment after COVID-19 infection were more likely to have fewer years of education (OR [95% CI]: 0.80 [0.72, 0.89], p<0.001) and pre-existing cognitive impairment (11.5 [4.50, 29.2], p<0.001) compared to those without (Table 2). Females (2.69 [1.13, 6.36], p = 0.03), individuals with a higher number of health conditions (1.34 [1.05, 1.70], p = 0.02), and those reporting functional limitation before COVID-19 (20.4 [8.36, 51.6], p<0.001) were more likely to have functional limitation after COVID-19 (Table 2). Pre-existing depressive symptoms (6.02 [2.60, 13.96], p<0.001) were associated with higher depressive symptoms after COVID-19 (Table 2). The number of nights in the hospital due to COVID-19 (0.84 [0.73, 0.97], p = 0.02) and total wealth over $125,000 (0.27 [0.11, 0.71], p = 0.008) were inversely associated with high depressive symptoms after COVID-19 (Table 2).

**Table 2:** Effects of demographics and number of health conditions on cognitive impairment, functional limitation, and high depressive symptoms among COVID-19 positive respondents ^†^.

	Post- COVID Cognitive Impairment (n = 260)		Post- COVID Functional limitation(n = 312)		Post- COVID High Depressive symptoms(n = 287)	
	OR (95% CI)	P Value	OR (95% CI)	P Value	OR (95% CI)	P Value
**Age**	1.03 (0.98, 1.09)	0.28	1.05 (1.00, 1.10)	0.07	1.03 (0.98, 1.07)	0.24
**Female**	0.61 (0.27, 1.38)	0.23	2.69 (1.13, 6.36)	0.03	1.08(0.52, 2.23)	0.84
**White Race**	0.42 (0.18, 0.97)	0.04	1.22 (0.55, 2.69)	0.60	0.99 (0.49, 1.98)	0.97
**Years of Education**	0.80 (0.72, 0.89)	<0.001	1.02(0.91, 1.14)	0.75	0.95 (0.86, 1.04)	0.26
**Married or partnered**	2.06 (0.77, 5.48)	0.15	1.08 (0.47, 2.48)	0.86	1.33 (0.62, 2.84)	0.47
**Total wealth ($)**						
<25 000	Reference		Reference		Reference	
25000–124999	0.57 (0.20, 1.65)	0.30	0.47 (0.17, 1.28)	0.14	0.85 (0.38, 1.91)	0.70
≧125000	0.81 (0.29, 2.28)	0.69	0.46 (0.17, 1.25)	0.13	0.27 (0.11, 0.71)	0.008
**Insurance**						
No insurance	Reference		Reference		Reference	
Federal Insurance	0.92 (0.23, 3.60)	0.90	0.33 (0.09, 1.14)	0.08	0.72 (0.25, 2.07)	0.54
Private Insurance	0.78 (0.23, 2.58)	0.68	0.32 (0.10, 1.05)	0.06	0.51 (0.19, 1.35)	0.17
Federal + Private Insurance	0.56 (0.10, 2.99)	0.50	0.44 (0.10, 1.88)	0.27	0.43 (0.11, 1.70)	0.23
**Number of Health Conditions**	0.98 (0.75, 1.27)	0.86	1.34 (1.05, 1.70)	0.02	1.18 (0.95, 1.48)	0.14
**Hospitalized between 2018 and 2020**	1.44 (0.56, 3.70)	0.45	1.91 (0.83, 4.41)	0.13	2.14 (0.97, 4.73)	0.06
**Number of nights in hospital due to COVID-19**	0.97 (0.89, 1.07)	0.59	1.00 (0.93, 1.08)	0.90	0.84 (0.73, 0.97)	0.02
**Time since COVID-19 infection** ^‡^	0.94 (0.81, 1.10)	0.46	1.02 (0.87, 1.19)	0.85	0.98 (0.86, 1.12)	0.80
**Baseline Cognitive Impairment**	11.5 (4.50, 29.2)	<0.001				
**Baseline Functional limitation**			20.5 (8.13, 51.6)	<0.001		
**Baseline High Depressive symptoms**					6.02 (2.60, 13.96)	<0.001

^†^ Each column represents one model. Results were calculated from logistic regression models, Odds Ratio (95% Confidence interval)

^‡^Time since COVID-19 infection using the time of the HRS 2020 survey completed less the time of the positive COVID-19 test.

### Symptoms and disabilities between COVID-19 positive individuals versus negative

Adjusting for demographic, pre-COVID-19/baseline health conditions, pre-COVID-19/baseline cognition, function, depressive symptoms, and hospitalization between 2018–2020, respondents who reported being COVID-19 positive had higher odds of any functional limitation (OR [95% CI]: 1.78 [1.26, 2.51], p = 0.001) and depressive symptoms (1.41 [1.04, 1.91], p = 0.03) compared to those who reported being COVID-19 negative. However, there was no significant association with cognitive impairment (1.11 [0.79, 1.55], p = 0.56) (see Table 3 and S2 Table in S1 File).

**Table 3:** Associations between COVID-19 infection and cognitive impairment, functional limitation, and high depressive symptoms in the crude analysis, multivariable analysis, and propensity-score matching.

		COVID-19 Positive	
		OR (95% CI)	P Value
**Cognitive Impairment** post-COVID-19/ follow-up	Crude model	0.98 (0.76, 1.28)	0.91
	Multivariable Analysis *	1.11 (0.79, 1.55)	0.56
	Propensity score matching ^‡^	1.39 (0.90, 2.13)	0.13
**Functional Limitation** post-COVID-19/ follow-up	Crude model	1.50 (1.18, 1.90)	0.001
	Multivariable Analysis *	1.78 (1.27, 2.51)	0.001
	Propensity score matching ^‡^	1.45 (1.02, 2.07)	0.04
**High Depressive symptoms** post-COVID-19/ follow-up	Crude model	1.38 (1.07, 1.78)	0.01
	Multivariable Analysis *	1.41 (1.04, 1.91)	0.03
	Propensity score matching ^‡^	1.94 (1.28, 2.94)	0.002

* Shown is the odds ratio from the multivariable logistic regression models, with adjustment for age, race, insurance, years of education, total wealth, number of health conditions, hospitalization between 2018–2020, pre-COVID-19/baseline cognition, functional limitation, and depressive symptoms. The analysis included 6075, 8750, and 8295 respondents in cognitive impairment, functional limitation, and high depression symptoms models, respectively.

^‡^ Shown is the odds ratio from the multivariable logistic regression models and covariates with matching according to the propensity score. The analysis included 323, 387, and 359 pairs in cognitive impairment, functional limitation, and high depression symptoms models, respectively.

Abbreviation: Odds Ratio (95% Confidence interval) = OR (95% CI)

Propensity-score matching yielded similar results (Table 3). The distribution of the estimated propensity scores for COVID-19 history in each functioning model is shown in S1 Fig in S1 File. The matched analytic sample pairs were 383, 387, and 359 in the cognitive impairment, functional limitation, and high depression symptom models, respectively. The standardized mean differences in the unmatched and matched sample are shown in S2 Fig in S1 File.

## Discussion

Using the HRS study, we compared post-COVID-19 cognition, functional status, and depressive symptoms in 409 COVID-19-positive individuals with their pre-illness baselines, along with 8,689 uninfected individuals. Among COVID-19-positive respondents, 24% exhibited new impairments after COVID-19 infection. These new impairments included a significant decrease in functional ability and a marginal decrease in cognitive function following COVID-19 infection when compared to individuals’ pre-infection baseline. When compared to 8,689 COVID-19-negative individuals, COVID-19-positive individuals had a significant decline in functional abilities and a high prevalence of depressive symptoms. Factors associated with impairments after COVID-19 infection included pre-existing impairments, less education, female gender, lower wealth status, prior hospitalization, higher comorbidity at baseline, and experiencing mild COVID-19 infection. Many COVID-19-positive respondents experienced PCC without awareness, especially older individuals with fewer pre-existing health conditions.

Previous research has focused on the prevalence of post-COVID-19 cognitive impairment through follow-up assessments without accounting for baseline cognitive function. These studies reveal that hospitalized COVID-19 patients experience significant cognitive impairment compared to non-infected controls [28–30]. Conversely, mild COVID-19 cases (non-hospitalized) do not demonstrate a significant cognitive impairment in comparison to non-infected controls [31]. This approach makes it difficult to determine the specific contribution of COVID-19 to incident cognitive impairment. After factoring in baseline cognitive functioning, our study discovered a marginal decrease in cognitive performance among COVID-19-positive respondents following infection. However, similar to the prior work, there was no significant decline in cognitive function among individuals with mild COVID-19 infection when compared to non-infected individuals. This suggests that the development of cognitive impairment after COVID-19 may be attributed to factors extending beyond the viral, encompassing variables such as level of education and pre-existing cognitive vulnerabilities.

Consistent with previous studies [3, 32–36], our research confirms that individuals, regardless of hospitalization status, experience new impairments in ADL/IADL after COVID-19 infection. These impairments were consistently observed in both within-group and between-group analyses, reaffirming the negative impact of COVID-19 on functional abilities. Such increased care dependency negatively impacts the quality of life for individuals and places a burden on caregivers [37, 38]. Additionally, our study findings align with other studies [5, 31, 39–42] showing that individuals with COVID-19 have significantly higher levels of depressive symptoms compared to healthy individuals. However, our study did not find significant differences in levels of depressive symptoms before and after infection among COVID-19-positive individuals. Therefore, further investigation is needed to determine whether the increased occurrence of high levels of depressive symptoms after COVID-19 infection is a consequence of the infection or the pandemic’s social and economic impacts [5]. This consideration is particularly relevant, given that the observed total wealth exceeding $125,000 was inversely associated with high levels of depressive symptoms following COVID-19 in our study.

Our findings highlight a significant discrepancy between objective measures and subjective awareness of PCC, which aligns with previous studies [6, 43–46]. Significantly, a noteworthy portion of individuals in our study displayed PCC Signs/symptoms without realizing they were experiencing PCC. This emphasizes the importance of incorporating both objective and subjective measurements to assess PCC. By integrating these approaches, we can gain a better understanding of the overall impact of COVID-19 on health. Additionally, our study indicates that age and pre-existing health conditions may influence an individual’s perception and awareness of their PCC. Further investigation is needed to elucidate the underlying factors contributing to these associations, including cognitive biases, psychological resilience, and social determinants of health. Such knowledge can help identify individuals who may be at a higher risk of underreporting or overlooking health effects after COVID-19 infection. It also enables the provision of necessary care, support, and interventions to ensure optimal recovery and well-being during the post-COVID-19 journey.

Finally, our study, consistent with previous research [12, 47–49], validates that individuals with milder COVID-19 symptoms (fewer nights in the hospital), lower levels of education, female gender, previous hospitalization, higher comorbidity, and lower wealth status are at a higher risk of developing PCC after infection. This underscores the importance of identifying the diverse care needs of individuals affected by COVID-19, especially for those with milder COVID symptoms, poor baseline health, and low socioeconomic status.

### Strengths and limitations

Our study exhibits several notable strengths. Firstly, we explore the prevalence of PCC in conjunction with its associated risk factors utilizing a nationally representative cohort. Furthermore, our analysis integrates measurements captured both prior to and subsequent to COVID-19 infection, representing an innovative approach. The robustness of our findings is additionally fortified through the utilization of both multivariable logistic regression and propensity score matching techniques. Nonetheless, our study does have several limitations. First, the HRS was not specifically designed to investigate PCC, thus commonly reported symptoms associated with PCC, such as post-exertional malaise and fatigue, were not captured. Additionally, the neuropsychological battery primarily assessed overall cognitive function, and may not have fully examined specific cognitive domains or enabled definitive clinical diagnoses. Despite employing cutoff scores associated with clinical dementia [50], our methodology may not have been sensitive enough to detect subtle or specific changes in cognitive abilities [51]. Furthermore, we excluded participants who were unable to complete the neuropsychological battery, potentially underestimating the true impact of COVID-19 on cognitive impairment. Moreover, our study primarily included individuals with mild COVID-19 infections, as only a small proportion were hospitalized. This suggests that the lack of observed cognitive impairment between COVID-19-infected and non-infected individuals in our sample may be attributed to the mild nature of the COVID-19 cases in our study population. Given that the majority of our study participants are aged 50 or older, a demographic more susceptible to various health concerns, additional research is needed to ascertain whether the noted impairments in our study are attributable to aging, other health conditions, or potential COVID-19 side effects.

## Conclusion

Within this nationally representative cohort of community-dwelling adults, 24% of COVID-19-positive respondents developed new impairments following their infection. COVID-19 infection was associated with increased odds of new functional limitation and high depressive symptoms but not cognitive impairment compared to those without COVID-19 infection. Nevertheless, among those with COVID-19 infection, declines in both functional ability and cognition were evident in comparison to their pre-COVID baselines. The varied outcomes observed in both within-person and between-person analyses of depressive symptoms suggest that the causes of post-COVID-19 depressive symptoms may extend beyond the direct effects of the virus. Psychological, social, and environmental factors may also contribute to the development of these symptoms. Therefore, further investigation is required to comprehensively understand the origins of these conditions. Additionally, comprehensive screening, encompassing both subjective and objective measures for PCC, is essential as many individuals may remain unaware of these deficits.

## Supporting information

S1 File (DOCX)

S1 Checklist STROBE statement—Checklist 1 of items that should be included in reports of observational studies. (PDF)
