# Supplementary material for: Cognition, function, and mood post-COVID-19: Comparative analysis using the health and retirement study
Source: PLoS One. 2024 Dec 18;19(12):e0315425. doi: 10.1371/journal.pone.0315425 (PMC11654941; doi:10.1371/journal.pone.0315425)
Supplement: S1 File — (DOCX) [file pone.0315425.s001.docx]

| **Table S1 Univariable logistic regression of demographic, baseline cognition, function, and depressive symptoms on self-report COVID history** | | |
| --- | --- | --- |
| **Variables** | **Self-reported COVID-19 Positive** | |
|  | **OR (95% CI)** | **p^£^** |
| Age, Median (IQR), y | 0.96 (0.95, 0.97) | <0.001 |
| Female, No. (%) | 0.94 (0.79, 1.13) | 0.53 |
| White Race, No. (%) | 0.71 (0.59, 0.85) | <0.001 |
| Education, Median (IQR), y | 0.97 (0.95, 0.99) | 0.03 |
| Married or partner, No. (%) | 1.26 (1.02, 1.55) | 0.03 |
| Total wealth ($), No. (%) |  |  |
| <25 000 | Reference |  |
| 25000-124999 | 0.83 (0.60, 1.12) | 0.24 |
| ≧125000 | 0.73 (0.59, 0.92) | 0.005 |
| Insurance, No. (%) |  |  |
| No insurance | Reference |  |
| Federal Insurance | 0.44 (0.32, 0.62) | <0.001 |
| Private Insurance | 0.83 (0.60, 1.15) | 0.25 |
| Federal + Private Insurance | 0.45 (0.31, 0.67) | <0.001 |
| Number of Health Conditions, Median (IQR), ^‡^ | 0.94 (0.88, 1.00) | 0.07 |
| Hospitalizations between 2018-2020, No. (%) | 1.54 (1.26, 1.87) | <0.001 |
| Baseline Cognitive Impairments, No. (%) ^¶^ | 0.88 (0.66, 1.16) | 0.39 |
| Baseline Functional limitations, No. ^§^ | 1.38 (1.05, 1.78) | 0.02 |
| Baseline High Depressive symptoms, No.  ^‖^ | 1.27 (0.97, 1.65) | 0.07 |
| **^†^ Each row represents one model. Results were calculated from logistic regression models.**  **^‡‡^A sum of whether the respondent has or had high blood pressure or hypertension; diabetes or high blood sugar; cancer or a malignant tumor of any kind except skin cancer; chronic lung disease except asthma such as chronic bronchitis or emphysema; heart attack, coronary heart disease, angina, congestive heart failure, or other heart problems; stroke or transient ischemic attack; emotional, nervous, or psychiatric problems; arthritis or rheumatism; Dementia or Alzheimer's disease - range from 0 to 9.**  **^¶^A 27-point scale was administered that included tests of memory, serial 7 subtractions, and backward counting. Cognitive impairment is defined as a total score <12.**  **^§^A sum of ADLs and IADLs, with a score ≥1, is defined as physical impairment.**  **^‖^An 8 -points scale measured by CES-D-8, with scores ≥4, is defined as having high depressive symptoms.**  **Abbreviation: Odds Ratio (95% Confidence interval) = OR (95% CI)** | | |

**Figure S1. Estimated propensity scores for self-report COVID-19 history in each functioning model**

**Figure S2. Standardized mean differences in the unmatched and matched sample in each functioning model.**

| **Table S2. Effect of demographic, number of health conditions, and COVID-19 history on cognitive impairments, functional limitations, and high depressive symptoms ^†^ (n=9,098)** | | | | | | |
| --- | --- | --- | --- | --- | --- | --- |
|  | **Cognitive Impairments in 2020** | | **Functional Limitations in 2020** | | **High Depressive symptoms in 2020** | |
|  | **OR (95 % CI)** | **P Value** | **OR (95 % CI)** | **P Value** | **OR (95 % CI)** | **P Value** |
| **COVID19 positive** | 1.11 (0.79, 1.55) | 0.56 | 1.78 (1.27, 2.51) | 0.001 | 1.41 (1.04, 1.91) | 0.03 |
| **Age** | 1.05 (1.04, 1.05) | <0.001 | 1.03 (1.02, 1.04) | <0.001 | 0.98 (0.97, 0.98) | <0.001 |
| **Female** | 0.81 (0.70, 0.94) | 0.005 | 1.35 (1.15, 1.58) | <0.001 | 1.30 (1.13, 1.51) | <0.001 |
| **White Race** | 0.65 (0.56, 0.76) | <0.001 | 1.10 (0.93, 1.31) | 0.25 | 1.12 (0.97, 1.30) | 0.13 |
| **Years of Education** | 0.89 (0.87, 0.91) | <0.001 | 0.97 (0.95, 0.99) | 0.03 | 0.95 (0.93, 0.97) | <0.001 |
| **Married or partnered** | 0.77 (66, 0.90) | 0.001 | 0.97 (0.82, 1.14) | 0.69 | 0.89 (0.77, 1.04) | 0.14 |
| **Total wealth ($)** |  |  |  |  |  |  |
| <25 000 | Reference |  | Reference |  | Reference |  |
| 25000-124999 | 0.86 (0.71, 1.03) | 0.10 | 0.94 (0.76, 1.15) | 0.55 | 0.95 (0.79, 1.15) | 0.60 |
| ≧125000 | 0.52 (0.43, 0.63) | <0.001 | 0.68 (0.55, 0.83) | <0.001 | 0.82 (0.68, 0.99) | 0.04 |
| **Insurance** |  |  |  |  |  |  |
| No insurance | Reference |  | Reference |  | Reference |  |
| Federal Insurance | 0.91 (0.69, 1.18) | 0.48 | 0.92 (0.68, 1.24) | 0.58 | 1.20 (0.93, 1.54) | 0.17 |
| Private Insurance | 0.71 (0.53, 0.94) | 0.02 | 0.42 (0.30, 0.60) | <0.001 | 0.69 (0.53, 0.89) | 0.005 |
| Federal + Private Insurance | 0.69 (0.50, 0.94) | 0.02 | 0.80 (0.57, 1.12) | 0.19 | 1.00 (0.74, 1.35) | 0.98 |
| Number of Health Conditions, Median ^‡^ | 1.08 (1.03, 1.14) | 0.001 | 1.34 (1.28, 1.41) | <0.001 | 1.25 (1.20, 1.31) | <0.001 |
| Hospitalizations between 2018-2020 | 1.20 (1.02, 1.44) | 0.03 | 1.93 (1.65, 2.26) | <0.001 | 1.29 (1.11, 1.51) | 0.001 |
| Baseline Cognitive Impairments^¶^ | 7.95 (6.82, 9.27) | <0.001 |  |  |  |  |
| Baseline Functional limitations ^§^ |  |  | 15.7 (13.4, 18.5) | <0.001 |  |  |
| Baseline High Depressive symptoms^‖^ |  |  |  |  | 8.77 (7.57, 10.2) | <0.001 |
| **^†^ Each column represents one model. Results were calculated from multivariable logistic regression models, Odds Ratio (95 % Confidence Interval [CI])**  **^‡‡^A sum of whether the respondent has or had high blood pressure or hypertension; diabetes or high blood sugar; cancer or a malignant tumor of any kind except skin cancer; chronic lung disease except asthma such as chronic bronchitis or emphysema; heart attack, coronary heart disease, angina, congestive heart failure, or other heart problems; stroke or transient ischemic attack; emotional, nervous, or psychiatric problems; arthritis or rheumatism; Dementia or Alzheimer's disease - range from 0 to 9.**  **^¶^A 27-point scale was administered that included tests of memory, serial 7 subtractions, and backward counting. Cognitive impairment is defined as a total score <12.**  **^§^A sum of ADLs and IADLs, with a score ≥1, is defined as physical impairment.**  **^‖^An 8 -points scale measured by CES-D-8, with scores ≥4, is defined as having high depressive symptoms.**  **Abbreviation: Odds Ratio (95% Confidence interval) = OR (95% CI)** | | | | | | |
