# Supplementary material for: Cognition, function, and mood post-COVID-19: Comparative analysis using the health and retirement study
Source: PLoS One. 2024 Dec 18;19(12):e0315425. doi: 10.1371/journal.pone.0315425 (PMC11654941; doi:10.1371/journal.pone.0315425)
Supplement: S1 Checklist — (PDF) [file pone.0315425.s002.pdf]

STROBE Statement—checklist of items that should be included in reports of observational studies

	Item No	Recommendation	Page No
**Title and abstract**	1	(*a*) Indicate the study's design with a commonly used term in the title or the abstract	P1
		(*b*) Provide in the abstract an informative and balanced summary of what was done and what was found	P1
**Introduction**			
Background/rationale	2	Explain the scientific background and rationale for the investigation being reported	P3
Objectives	3	State specific objectives, including any prespecified hypotheses	P3
**Methods**			
Study design	4	Present key elements of study design early in the paper	P4
Setting	5	Describe the setting, locations, and relevant dates, including periods of recruitment, exposure, follow-up, and data collection	P4
Participants	6	(*a*) *Cohort study*—Give the eligibility criteria, and the sources and methods of selection of participants. Describe methods of follow-up<br>*Case-control study*—Give the eligibility criteria, and the sources and methods of case ascertainment and control selection. Give the rationale for the choice of cases and controls<br>*Cross-sectional study*—Give the eligibility criteria, and the sources and methods of selection of participants	P4,5
		(*b*) *Cohort study*—For matched studies, give matching criteria and number of exposed and unexposed<br>*Case-control study*—For matched studies, give matching criteria and the number of controls per case	P4,5,6
Variables	7	Clearly define all outcomes, exposures, predictors, potential confounders, and effect modifiers. Give diagnostic criteria, if applicable	P4,5
Data sources/ measurement	8*	For each variable of interest, give sources of data and details of methods of assessment (measurement). Describe comparability of assessment methods if there is more than one group	P4,5
Bias	9	Describe any efforts to address potential sources of bias	P4,5, 12
Study size	10	Explain how the study size was arrived at	Figure1
Quantitative variables	11	Explain how quantitative variables were handled in the analyses. If applicable, describe which groupings were chosen and why	P6-7
Statistical methods	12	(*a*) Describe all statistical methods, including those used to control for confounding	P6-7
		(*b*) Describe any methods used to examine subgroups and interactions	P6-7
		(*c*) Explain how missing data were addressed	P6-7
		(*d*) *Cohort study*—If applicable, explain how loss to follow-up was addressed<br>*Case-control study*—If applicable, explain how matching of cases and controls was addressed<br>*Cross-sectional study*—If applicable, describe analytical methods taking account of sampling strategy	P6-7
		(*e*) Describe any sensitivity analyses	

**Results**			
Participants	13*	(a) Report numbers of individuals at each stage of study—eg numbers potentially eligible, examined for eligibility, confirmed eligible, included in the study, completing follow-up, and analysed	P7
		(b) Give reasons for non-participation at each stage	Figure1
		(c) Consider use of a flow diagram	Figure1
Descriptive data	14*	(a) Give characteristics of study participants (eg demographic, clinical, social) and information on exposures and potential confounders	P7
		(b) Indicate number of participants with missing data for each variable of interest	Table1
		(c) *Cohort study*—Summarise follow-up time (eg, average and total amount)	P7
Outcome data	15*	*Cohort study*—Report numbers of outcome events or summary measures over time	
		Case-control study—Report numbers in each exposure category, or summary measures of exposure	P7
		Cross-sectional study—Report numbers of outcome events or summary measures	
Main results	16	(*a*) Give unadjusted estimates and, if applicable, confounder-adjusted estimates and their precision (eg, 95% confidence interval). Make clear which confounders were adjusted for and why they were included	P7-9
		(*b*) Report category boundaries when continuous variables were categorized	P7-9
		(*c*) If relevant, consider translating estimates of relative risk into absolute risk for a meaningful time period	P7-9
Other analyses	17	Report other analyses done—eg analyses of subgroups and interactions, and sensitivity analyses	P7-9
**Discussion**			
Key results	18	Summarise key results with reference to study objectives	P9
Limitations	19	Discuss limitations of the study, taking into account sources of potential bias or imprecision. Discuss both direction and magnitude of any potential bias	P11
Interpretation	20	Give a cautious overall interpretation of results considering objectives, limitations, multiplicity of analyses, results from similar studies, and other relevant evidence	P12
Generalisability	21	Discuss the generalisability (external validity) of the study results	P11
**Other information**			
Funding	22	Give the source of funding and the role of the funders for the present study and, if applicable, for the original study on which the present article is based	P13

*Give information separately for cases and controls in case-control studies and, if applicable, for exposed and unexposed groups in cohort and cross-sectional studies.

Note: An Explanation and Elaboration article discusses each checklist item and gives methodological background and published examples of transparent reporting. The STROBE checklist is best used in conjunction with this article (freely available on the Web sites of PLoS Medicine at http://www.plosmedicine.org/, Annals of Internal Medicine at http://www.annals.org/, and Epidemiology at http://www.epidem.com/). Information on the STROBE Initiative is available at www.strobe-statement.org.
